# Effect of Zinc on Improving Silver Diamine Fluoride-derived Tooth Discoloration in Vitro

**DOI:** 10.21203/rs.3.rs-5078856/v1

**Published:** 2024-11-13

**Authors:** Abdullah Almulhim, Astrid C. Valdivia-Tapia, Guilherme Roncari Rocha, Yan Wu, Xinyue Mao, Nora Alomeir, Danielle Benoit, Anderson T. Hara, Tong Tong Wu, Jin Xiao, Yihong Li

**Affiliations:** University of Rochester Medical Center; Indiana University School of Dentistry; University of Oregon; University of Rochester Medical Center; University of Rochester Medical Center; University of Rochester Medical Center; University of Oregon; Indiana University School of Dentistry; University of Rochester Medical Center; University of Rochester Medical Center; Cornell University College of Veterinary Medicine

**Keywords:** Silver Diamine Fluoride, Zinc, Tooth Discoloration, Dentin, Caries Treatment

## Abstract

**Background:**

Silver Diamine Fluoride (SDF) is effective for arresting dental caries, presenting a valuable non-invasive treatment option in dentistry. Despite its therapeutic advantages, a significant drawback is the tooth discoloration that follows its application, which can affect patient acceptance. Addressing this aesthetic concern without diminishing the treatment’s efficacy remains challenging in dental practice. This study explores strategies to improve the aesthetic outcomes of SDF treatments.

**Methods:**

This *in vitro* study assessed the efficacy of Zinc in reducing SDF-induced discoloration on dentin blocks and examined its impact on the physical properties of dentin, including hardness and roughness. Dentin blocks were pre-treated with various concentrations of Zinc, followed by SDF application. Color changes were analyzed using Image J software, and cytotoxicity was evaluated using the CytoTox-ONE^™^ Homogeneous Membrane Integrity Assay. Dentin surface characteristics, including micro-hardness and roughness, were assessed using scanning electron microscopy.

**Results:**

The study results revealed a dose-dependent efficacy of Zinc in reducing discoloration caused by SDF on dentin, with higher Zinc concentrations showing better improvement in color outcomes. The application of a 20M Zinc solution prior to SDF treatment significantly reduced discoloration compared to SDF alone, measured on day 14. Additionally, no significant changes in the hardness or roughness of etched dentin were observed in Zinc + SDF group compared to the SDF alone group. Zinc treatments demonstrated a desirable outcome on mucosal cytotoxicity, comparable to that of the negative control.

**Conclusion:**

Zinc significantly reduced SDF-induced tooth discoloration in a dose-dependent manner without affecting the etched dentin’s micro-hardness and roughness, potentially improving patient acceptance, especially in visible areas of the mouth. Further research is warranted to validate the effectiveness of this zinc-enhanced treatment protocol *in vivo*.

## Introduction

Dental caries is a significant global health challenge that affects individuals of all ages, with untreated caries in permanent teeth being the most common health condition worldwide (1). Early childhood caries (ECC) is a particularly aggressive form of this disease, affecting children under six and significantly impacting their quality of life (2). ECC is recognized as a major public health issue, affecting nearly half of preschool-aged children worldwide, leading to significant health and economic consequences if left untreated (3). Despite the availability of treatments, the unique challenges presented by ECC, especially in underserved communities, highlight the need for new, non-invasive treatment options (4).

Silver diamine fluoride (SDF) has been recognized for its potential to arrest dental caries over the past decades, offering a promising non-invasive treatment option for ECC. Evidence supports SDF’s effectiveness in caries prevention and arrest (6). SDF works through the antimicrobial action of silver against cariogenic bacteria and fluoride’s promotion of tooth enamel remineralization (7). However, SDF application is associated with compromised tooth aesthetics following treatment. This SDF-derived discoloration, which can range from mild to severe black staining, results from silver’s reaction with dental tissues (4). Studies have indicated that while dental professionals and patients recognize the efficacy of SDF in caries management, the potential for noticeable staining can deter its use, especially in visible areas of the mouth (8). Patient perception of this side effect significantly influences treatment decisions, underscoring the need for strategies to minimize discoloration without diminishing SDF’s therapeutic benefits (9).

Various strategies have been Employed to address the issue to reduce the discoloration associated with SDF treatment. A notable approach is immediately applying a saturated potassium iodide (KI) solution after SDF administration. This leads to a reaction between KI and silver ions, resulting in the formation of silver iodide, which is less visible. While SDF/KI exhibited lower discoloration intensity than SDF alone, the difference was not statistically significant (10). An alternative technique, referred to as the Smart technique, involves the post-SDF application of a thin glass ionomer cement (GIC) layer on the tooth surface. GIC acts as a barrier, encapsulating the SDF and minimizing its exposure to oral substances that could cause discoloration (11). However, study findings also suggest that while KI and the Smart technique may offer some reduction in SDF-induced discoloration, they do not provide complete elimination of this issue. Further research is required to develop more effective methods for minimizing SDF-related discoloration (9, 12).

Zinc, known for its antimicrobial properties and potential to enhance remineralization, has emerged as a promising candidate to improve the aesthetic outcomes of SDF treatment without compromising its therapeutic efficacy (7). Thus, this study aims to assess the efficacy of Zinc in reducing SDF-induced discoloration of dentin blocks *in vitro* and its impact on the physical properties of the dentin surface.

## Methods

### Dentin block preparation

**Figure 1:**
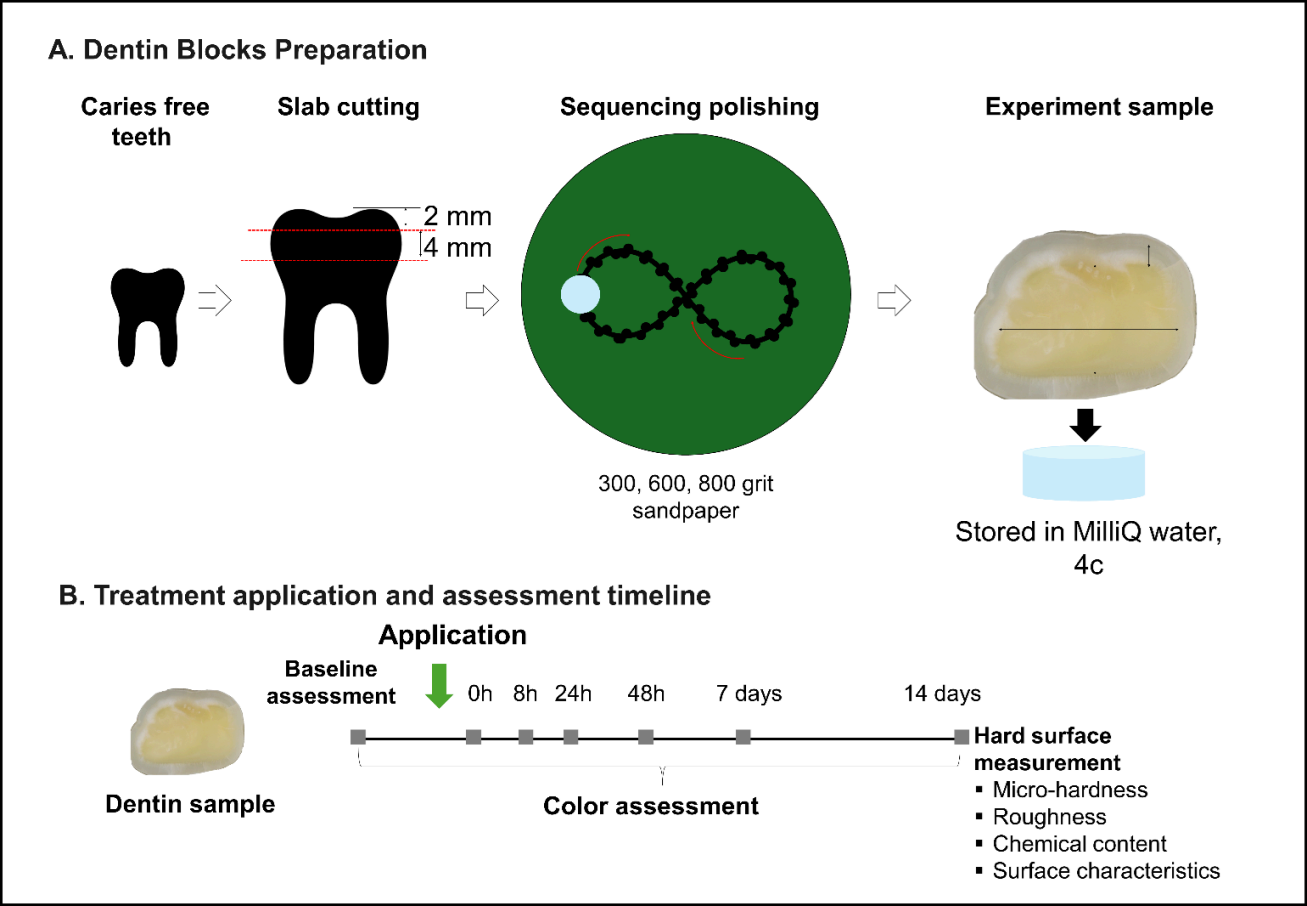
Study Design and Assessment Timeline. Figure 1. Sample preparation and Overview of Study Design and Assessment Timeline: This timeline illustrates the sequential steps involved in dentin block preparation (A) HA discs preparation, treatment application, and assessment schedule (B) Experimental timeline, providing a visual guide to the experimental workflow.

The dentin preparation steps are illustrated in Fig. 1. Human permanent teeth (premolars and molars), free from caries and dental restorations, were selected for this study. Tooth samples were sectioned to isolate the crown portion, then precisely cut into 3–4mm thick dentin blocks using a Techcut 4^™^ (Allied Equipment and Fixtures, USA). This step ensured uniformity across all samples, which is crucial for the study’s consistency. Post-sectioning, dentin blocks underwent a multi-stage polishing process using progressively finer grits of sandpaper (300, 600, and 800) to attain a smooth, uniform surface, eliminating any irregularities or debris that could influence treatment outcomes. Polished blocks were then stored in Milli-Q water at 4 °C. Milli-Q water was used to maintain hydration and preserve the structural integrity of the dentin.

### Zinc solution preparation

Zinc nitrate hexahydrate (Sigma-Aldrich, Saint Louis, MO, USA) was selected due to its high purity and solubility, facilitating consistent concentration levels across solutions. A wide range of Zinc concentrations was prepared for effective testing. The Zinc compound (molecular weight of approximately 297.49 g / mol) was dissolved in phosphate-buffered saline (PBS) to create Zinc solutions with concentrations ranging from 0.1M to 20M. After dissolution, solutions underwent rigorous homogenization to ensure uniform distribution of Zinc.

### Application of Zinc and SDF Treatment

The following groups were tested in the study: negative control (untreated), positive control (SDF only), and treatment groups receiving various Zinc concentrations before SDF application. For the positive control and treatment groups, each dentin block was first treated with 37% phosphoric acid etch gel (Prime Dental, Chicago, USA) using a micro brush for thirty seconds, then thoroughly washed with distilled water to prepare the surface. After this preparatory step, 25 microliters of Zinc solution were applied to each block in the treatment group using a micro brush, followed by the application of 38% SDF. The positive control received only the SDF application following the acid etch. Each treatment was meticulously applied for 1 minute to ensure consistent exposure across all samples. After the application of treatments, the treated dentin blocks were stored in an adsorption buffer within a 24-well culture plate (Corning Incorporated, Corning, NY, USA) at 37°C to maintain the hydration of dentin blocks. Three discs were used for each group at each experiment round, with three independent rounds being conducted.

### Tooth Color Assessment

**Figure 2:**
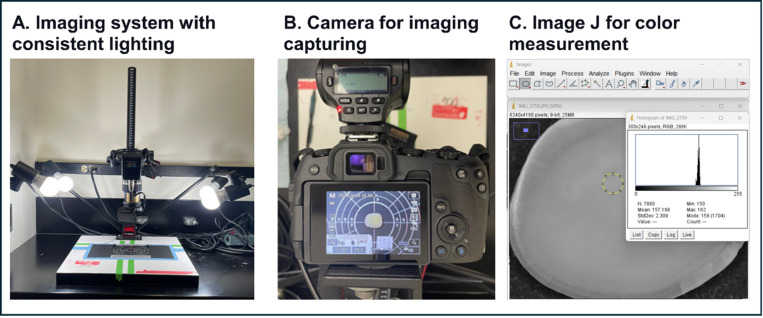
Imaging Setup for Color Change Documentation in Dentin Blocks Specialized Imaging Setup for Documenting Dentin Color Changes: Illustrates (A) the consistent lighting system used to standardize the illumination of samples throughout the color assessment process. (B) the high-resolution camera setup that captured detailed images of the dentin blocks, ensuring precise documentation of coloration changes post-treatment. (C) *is an example of the utilization of Image J software for color measurements, illustrating how the software quantifies color changes to provide objective data on the treatment effects*.

Color changes of the dentin blocks were assessed at the following time points: immediately after treatment and at 6 hours, 24 hours, 48 hours, 1 week, and 2 weeks post-application (Fig. 1). A specialized imaging setup, equipped with a consistent lighting system (Firenze Mini Repro/4 1194 Repro with Lights, Manfrotto, Cassola, Italy) and a Canon EOS RP camera (Canon Inc., Tokyo, Japan) equipped with a lens Tamron 90mm F/2.8 Di MACRO 1:1 VC USD (Tamron Co., Ltd., Saitama, Japan), was used to capture the coloration changes (Fig. 2). The histogram 8-bit feature in Image J software (U. S. National Institutes of Health, Bethesda, Maryland, USA) was used to measure color intensity variations at five specific points on each block.

### Epithelial Cells Safety Assessment

To evaluate the cytotoxic effects of Zinc (15M and 20M concentrations) and 38% SDF on oral mucosal cells, the squamous carcinoma of buccal mucosa-derived epithelial cell line TR-146 (Product #10032305, Millipore Sigma, Darmstadt, Germany) was utilized. Cells from passages 4 to 10 were cultured in DMEM/F12 medium (Gibco^™^ #11320033) supplemented with 10% v/v fetal bovine serum (FBS) (Gibco^™^ #16140071, Thermo Scientific^™^, Waltham, MA), in 25cm^2^ plastic flasks (VWR^®^ #10062872, Radnor, PA), without the addition of antibiotics or antifungal agents. The cells were incubated in a humidified atmosphere at 37°C and 5% CO2.

The CytoTox-ONE^™^ Homogeneous Membrane Integrity Assay (Promega Corporation, Madison, WI, USA) was employed for the cytotoxicity assessment. This fluorometric assay quantifies the release of lactate dehydrogenase (LDH) from cells, serving as an indicator of cell membrane integrity. The assay measures explicitly LDH release into the culture medium, using a coupled enzymatic reaction that converts resazurin into the fluorescent product resorufin. TR-146 cells were treated with 25 microliters of the designated concentrations of Zinc solutions (15M and 20M) and 38% SDF for 1 minutes, with PBS-treated cells acting as a negative control. Following the treatment, CytoTox-ONE^™^ Reagent was added to the culture medium, and after a 10-minute incubation, the fluorescence signal was measured to quantify LDH release. This measurement was performed using a 96-well microtiter plate reader (Infinite M200 PRO, Tecan).

### Tooth Surface Texture Analysis

Surface roughness parameters (Ra and Rq, in μm) were evaluated using a non-contact profilometer (Proscan 2000, Scantron Industrial Products Ltd., Taunton, England, UK) and dedicated software (version 2.0.17 Scantron Industrial Products Ltd, Taunton, England, UK). The analysis was confined to a 1×1mm^2^ area scanned with an S5/03 sensor at 10μm step size, with three independent measurements performed per specimen (13) in three locations under the cusps. Mean Ra and Rq values were calculated for each experimental group.

### Tooth Surface Microhardness Analysis

Dental specimens were mounted on acrylic blocks and tested for surface microhardness (Knoop Hardness Number, KHN) using a Knoop indenter under a 50g load with a 5-second dwell time (2100 HT, Wilson Instruments, Norwood, MA, USA). Five indentations were placed 100-μm apart from each other and in three locations under the cusps (14).

### Scanning Electron Microscopy (SEM) and Energy-Dispersive X-ray Spectroscopy (EDS)

Specimens were air-dried in a desiccator and coated with gold–palladium alloy to a 200 A° thickness (Denton vacuum Desk). They were analyzed in high vacuum conditions using a JEOL 7800F field-emission SEM (JEOL Ltd., Tokyo, Japan) at 20 Kv, 10 spot size, and a 10 mm working distance. Secondary electron images were taken from representative areas of each specimen at different magnifications (15).

Energy-dispersive X-ray spectroscopy (EDS) was performed using the same SEM setup to determine the elemental composition of the dentin surfaces. The EDS analysis focused on detecting key elements such as silver (Ag), zinc (Zn), phosphorus (P), and calcium (Ca) across different treatment groups.

### Statistical Analysis

The primary objective of our statistical analysis was to evaluate the efficacy of Zinc treatments in reducing dentin surface darkness and to identify the optimal Zinc concentration for this purpose. We employed simple linear regression and likelihood ratio tests to achieve this. The simple linear regression analysis assessed the relationship between treatment groups and tooth whiteness, using data from day 14 as the primary endpoint. The mean whiteness across five predetermined points on each tooth was the dependent variable, while treatment type and corresponding baseline whiteness acted as independent variables. This model provided initial insights into the treatment effects, albeit with simplified assumptions and reduced data complexity. Likelihood ratio tests evaluate the goodness of model fit and assess the statistical significance of the treatment effects. In the analysis of dentin surface microhardness and roughness, two-sample T-tests were exclusively employed to compare all treatment groups to the positive and negative controls, following assessments of data normality. This method facilitated focused comparisons to understand the specific effects of treatments relative to control conditions. All statistical analyses were performed using R software version 4.2.1 (R Foundation for Statistical Computing, Vienna, Austria).

## Results

### Effectiveness of Zinc in Reducing SDF-Induced Discoloration

**Figure 3:**
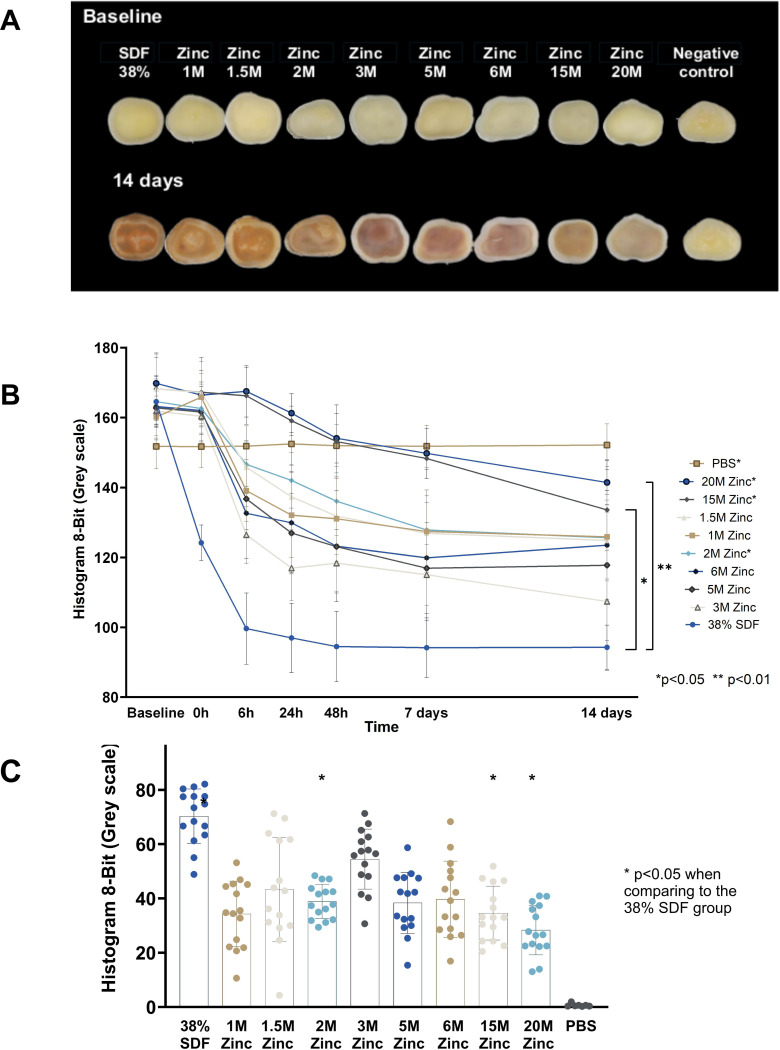
Dentin Block Color Change Over Time **(A)** Macro images from HA discs showing the discoloration in dentin blocks following treatment with various concentrations of Zinc and SDF, highlighting the correlation between treatment type and color intensity change. (B) Measurement of the grey scale from baseline to 14 days. (C) Delta changes of color (grey scale) between the baseline and Day 14. Statistical significance was detected between 38% SDF vs. 2M Zinc, 38% SDF vs. 15M Zinc, and 38% SDF vs. 20M Zinc (p<0.05).

The study results indicated a dose-dependent efficacy of Zinc in mitigating the effects of discoloration. While lower concentrations of Zinc showed limited capacity to prevent the darkening of the dentin samples, a clear trend emerged as the concentration increased. The application of 20M Zinc solution prior to SDF treatment significantly reduced discoloration (Fig. 3).

A Linear Regression model was employed to assess the effect of 20M Zinc, in conjunction with 38% SDF, on tooth whiteness. It was observed that baseline whiteness is a highly predictive covariate when modeling day 14 whiteness. However, there was ambiguity in defining baseline whiteness: whether to consider the mean whiteness among five spots before treatment or immediately after treatment. To address this, we introduced Δ, defined as the difference in mean whiteness between before and immediately after treatment, capturing some baseline properties of the teeth. In the Linear Regression model, three covariates—treatment, Δ, and initial whiteness before applying Zinc—were considered. The treatment significantly influenced the outcome variable, mean whiteness, on day 14. The teeth treated with 20M Zinc + 38% SDF exhibited a mean whiteness that was 30.76 units higher compared to those treated with 38% SDF alone, controlling for the other two variables. The likelihood ratio test yielded a p-value of 0.0058, indicating a statistically significant treatment effect, thus strongly suggesting the efficacy of 20M Zinc in enhancing tooth whiteness post-SDF treatment.

Furthermore, an analysis of the treatment effect pattern was conducted to identify the optimal concentration of Zinc. The histogram in Fig. 3 illustrates the change in whiteness among different treatment groups. The negative control group showed minimal whiteness change, while the positive control group experienced substantial darkening. The treatment group with higher Zinc concentrations showed less whiteness change, effectively preventing darkening. Further exploration with Simple Linear Regression models, considering treatments across a spectrum of Zinc concentrations, revealed that starting with the same initial whiteness and Δ, the day 14 whiteness was higher for teeth treated with various Zinc concentrations compared to the positive control (38% SDF alone). Specifically, the highest increase was 26.77 for the 20M Zinc treatment, with a p-value of 0.0019, followed by an 18.81 increase for the 15M Zinc treatment, with a p-value of 0.023, underscoring a concentration-dependent improvement in whiteness. Interestingly, Δ also showed a statistically significant effect on day 14 whiteness, with a coefficient of 0.54 and a p-value of 6.01×10^− 4^, indicating a concordant relationship between immediate and 14-day whiteness changes.

### Cytotoxicity of Zinc and SDF Treatments

The cytotoxicity of each treatment was quantified using the percent cytotoxicity formula, which takes into account the fluorescence values indicative of LDH release:

Percent Cytotoxicity = 100 × (Experimental – Culture Medium Background) / (Maximum LDH Release – Culture Medium Background)

**Figure 4:**
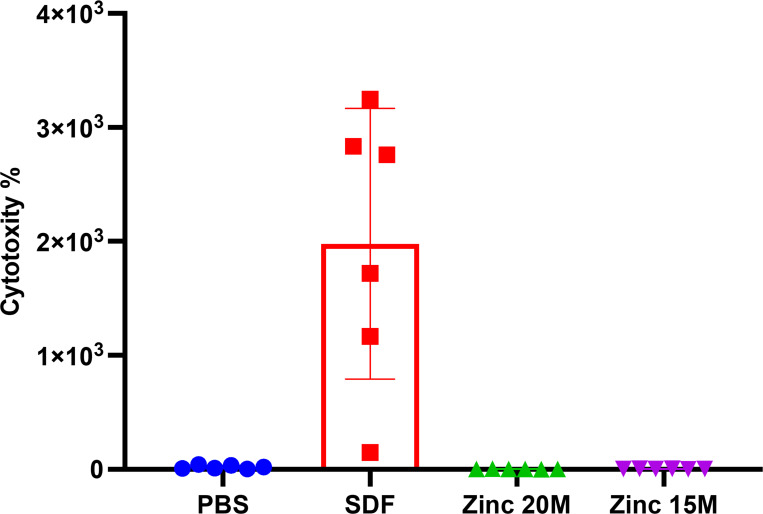
Safety Assessment Results Figure 4. Cytotoxicity Profiles of Treatments: Shows the comparative cytotoxic effects of different treatment groups on epithelial cells, illustrating the safety profile of Zinc in combination with SDF.

Our findings reveal contrasting cytotoxicity profiles between SDF and Zinc-treated groups. SDF alone displayed a notably high cytotoxicity towards epithelial cells, highlighting its potential adverse effects on cell membrane integrity. In contrast, treatments involving Zinc, particularly at concentrations of 15M and 20M, demonstrated significantly lower cytotoxicity levels than SDF. Notably, the 20M Zinc solution exhibited no evident toxicity on buccal mucosal cells, as depicted in Fig. 4.

### Dentin Surface Texture Analysis

**Figure 5:**
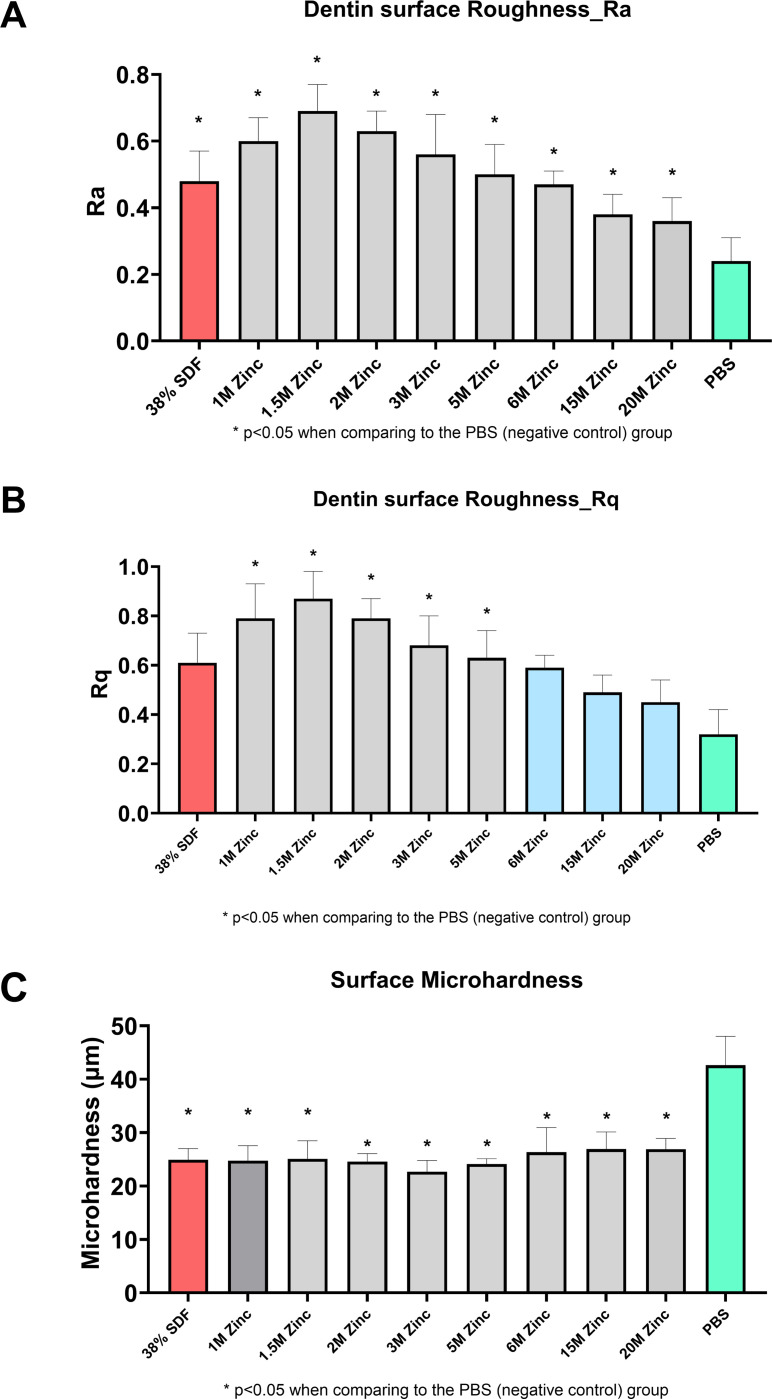
Evaluation of Zinc Concentration Effects on Dentin Surface Texture and Microhardness Post-SDF Treatment Figure 5. Presents (A-B) Surface roughness measurements (Ra and Rq) at varying Zinc concentrations (0.1M to 20M) show the impact of Zinc application compared to controls (negative control: untreated, positive control: 38% SDF). (C) Surface microhardness, expressed in Knoop Hardness Number (KHN), reveals the preservation or enhancement of dentin hardness with Zinc treatment. These results demonstrate Zinc’s dual ability to mitigate SDF-induced discoloration while maintaining or improving the mechanical properties of dentin, supporting Zinc’s potential integration into SDF treatments for optimized aesthetic and structural outcomes.

Etched dentin surface texture was evaluated through the roughness parameters Ra and Rq, and did not reveal a consistent Zinc dose-response relationship. Specimens treated with PBS (negative control) presented the lowest roughness values, differing from all other groups for Ra (Fig. 5a). For Rq, a similar pattern was observed, however, there was no significant difference between the negative control group and those treated with SDF, 6M, 15M, and 20M Zinc solutions (p > 0.05, Fig. 5b). This observation suggests that applying either Zinc or SDF may affect the dentin surface through mechanisms such as surface deposition.

### Dentin Surface Microhardness

All treated groups exhibited significantly reduced microhardness values compared to the negative control (p < 0.05). These observed values were markedly lower than the typical microhardness of sound dentin, which generally hovers around KHN 50. This widespread reduction in surface microhardness in the treated samples indicates a likely softening of the dentin surface, potentially due to demineralization effects induced by the applied treatments. Notably, the study protocol involved applying 37% phosphoric acid etch gel to sound dentin in all treatment groups—except the negative control—prior to treatment application. This preparatory step likely contributed to the pronounced decrease in microhardness observed in these groups, emphasizing the need to consider the implications of pre-treatment procedures on dentin’s mechanical integrity.

### SEM Observation

The SEM images of the dentin surface were obtained for one representative specimen from each group. The images indicated that dentin treated with SDF alone presented a superficial layer that largely obliterated the dentinal tubules. A similar pattern was observed in the PBS group, where dentinal tubule obliteration was most likely due to the presence of a smear layer, since these specimens were not acid-etched. In contrast, all groups treated with Zinc solutions showed total or partial exposure of the dentinal tubules, which became more evident in specimens treated with Zinc concentrations above 1M.

**Figure 6:**
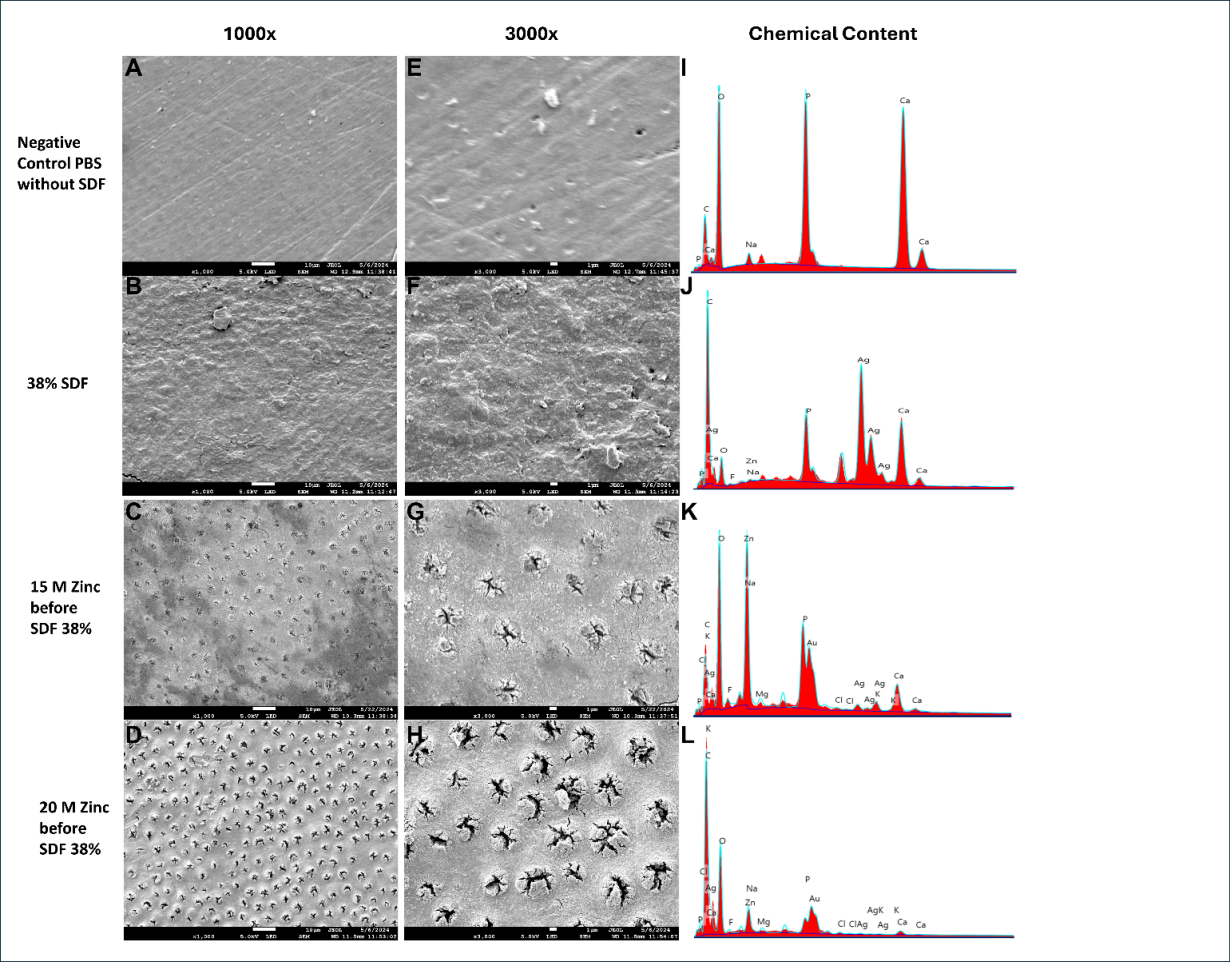
SEM and EDS Analysis of Dentin Surfaces Treated with Zinc and SDF: Figure 6 presents (A-D) SEM images at 1000x magnification show the surface morphology and degree of dentinal tubule exposure across different treatment conditions. (E-H) SEM images at 3000x magnification provide a closer look at the structural details, emphasizing the differences in surface texture. (I-L) Energy-dispersive X-ray spectroscopy (EDS) data reveal the elemental composition, indicating minimal Zinc presence in the SDF-only group and significant Zinc deposition in Zinc-treated groups. The displacement of silver by Zinc is evident, potentially explaining the reduction in discoloration observed in these groups.

Notably, energy-dispersive X-ray spectroscopy (EDS) analysis revealed minimal Zinc presence in the SDF-only group, while significant Zinc deposition was observed in groups treated with Zinc and SDF. The result suggests that Zinc may effectively replace silver on the dentin surface, potentially explaining the observed reduction in discoloration. The displacement of silver by Zinc in these groups underscores the role of Zinc in mitigating the staining effects typically associated with SDF, as Zinc appears to alter the deposition patterns of silver on the dentin surface (Fig. 6).

## Discussion

In this investigation, we explored the efficacy of Zinc in counteracting the undesired discoloration effects associated with the application of SDF for caries arrestment. Our findings demonstrated that 20M of Zinc significantly reduced the discoloration derived from SDF treatment. Clinically, incorporating Zinc into SDF treatments could enhance patient acceptance and broaden the utility of SDF, particularly in visible areas of the mouth where aesthetic concerns are paramount. From a public health perspective, improving the acceptability of SDF could lead to broader adoption of this cost-effective caries management strategy, especially in underserved communities where access to dental care is limited.

Another highlight is that we demonstrated a dose-dependent relationship between Zinc application and discoloration mitigation, with higher concentrations of Zinc correlating with more pronounced color improvement. This relationship is statistically significant and clinically relevant, suggesting a potential for customized treatment protocols based on patient-specific aesthetic concerns and treatment goals.

Moreover, our study investigated the impact of Zinc on the physical properties of the dentin surface, such as hardness and roughness, which are critical to the tooth’s structural integrity and function. The results indicate that applying Zinc, even at higher concentrations, does not compromise these properties. This finding is paramount, as it alleviates concerns regarding the potential for Zinc to adversely affect the tooth’s resilience or susceptibility to wear and subsequent caries development.

### Zinc’s features and safety

Zinc, an essential trace element, is pivotal in various biological processes, including enzyme function, immune response, and wound healing. Its application extends beyond dental care into areas such as dermatology, where Zinc oxide is commonly used for its protective and healing properties in skin care products and sunscreens (16). In nutrition, Zinc supplements are recognized for bolstering immune system function and have been utilized to manage common colds and other illnesses (17).

Zinc’s role in dentistry extends beyond its recent application in mitigating the aesthetic limitations of SDF treatment. It has long been recognized for its biomimetic properties, particularly in enhancing the remineralization of enamel and dentin (18). This trace element is a fundamental component of various dental materials, including cements and amalgams, where its inclusion is not merely for structural benefits but also for its capacity to promote dental tissue regeneration and caries resistance.

The biomimetic action of Zinc, especially in the form of Zinc hydroxyapatite, underscores its potential to closely mimic the natural processes of mineralization in dental tissues, thereby supporting the integrity and longevity of dental restorations and preventive treatments. The broad utilization of Zinc across these diverse fields underscores its biocompatibility and safety, a crucial consideration in developing any therapeutic or preventive agent.

The safety and importance of Zinc in oral health extend beyond its application in dental materials, reaching into its essential role in overall oral wellness. Uwitonze et al. (2020) emphasize that Zinc adequacy is crucial for maintaining optimal oral health, underscoring its significance not only as a component of dental treatments but also as a vital nutrient. The comprehensive review by Uwitonze and colleagues highlights the minimal adverse effects of Zinc, whether utilized systemically as a dietary supplement or locally in dental applications (19). This evidence consolidates the foundation for Zinc’s suitability in long-term oral health strategies, marking it as a key element in preventive and restorative dental care practices. This established safety record is crucial in our study, which proposes the application of Zinc as an adjunct to SDF treatment to mitigate tooth discoloration.

Importantly, our findings corroborate the well-established safety profile of Zinc. In our study, the application of Zinc across a range of concentrations did not exhibit cytotoxic effects on dental tissues. This aligns with the evidence supporting Zinc’s biocompatibility and reinforces the potential for its safe incorporation into dental treatment protocols, particularly those aimed at managing early childhood caries (ECC) with SDF.

### Outcome comparison to other approaches

The search for effective methods to mitigate the aesthetic compromise of SDF-induced tooth discoloration has led to exploring various strategies, with potassium iodide (KI) and the SMART technique being the most notable. While KI has shown potential in reducing discoloration through the formation of a less visible compound with silver ions, its efficacy in eliminating discoloration remains limited, as evidenced by studies from Detsomboonrat et al. (2022) and Lee et al. (2022). Similarly, the SMART technique, which employs glass ionomer cement to conceal discoloration, faces durability and fracture resistance challenges, potentially compromising long-term aesthetic outcomes (Chen et al., 2021; Sidhu & Nicholson, 2016).

Our study introduces Zinc as a novel and superior alternative, effectively addressing the limitations of KI and the SMART technique. Zinc’s interaction with SDF-treated dentin not only reduces the visibility of discoloration but does so through a mechanism that suggests an intrinsic alteration of the discoloration process itself. This unique approach, coupled with Zinc’s straightforward application, safety profile, and preservation of dentin’s hard surface properties, distinguishes it as a markedly effective solution for enhancing the aesthetic outcomes of SDF treatments.

By directly comparing Zinc to existing discoloration mitigation strategies, our findings reveal its potential to significantly enhance patient and clinician acceptance of SDF as a viable caries management strategy. Furthermore, the biomimetic potential of Zinc in supporting dental tissue remineralization and structural integrity, as Andrea et al. (2023) highlighted, reinforces its value in dental applications beyond discoloration mitigation.

In summary, Zinc offers a groundbreaking approach to overcoming one of the major aesthetic challenges associated with SDF treatment. It positions itself as an invaluable adjunct in the minimally invasive management of dental caries, with implications for improved patient satisfaction and treatment acceptance.

### Limitations

While pioneering its approach to mitigating SDF-induced tooth discoloration using Zinc, this study has the following limitations. Firstly, the *in vitro* nature of our experimental design and the relatively small sample size for some of the tests, although instrumental in establishing a foundational understanding of Zinc’s efficacy, may only partially replicate the complex interactions, conditions, and variations in the oral environment. Thus, translating these findings to clinical practice requires cautious optimism and further validation.

Another significant limitation concerns the preparatory use of 37% phosphoric acid etch gel on dentin surfaces. This step was essential for simulating the demineralized conditions under which SDF typically interacts with tooth structure, facilitating noticeable discoloration necessary for the aims of our study. However, this etching process may also alter the inherent properties of dentin, affecting measures of roughness and microhardness. These alterations could confound the assessment of Zinc’s actual effect on these physical properties, potentially overstating or understating the protective effects of Zinc. Future studies should consider these variables and explore methods to isolate the impact of each treatment component more distinctly. Another area for improvement lies in the scope of Zinc concentrations explored. While we identified a concentration-dependent relationship between Zinc application and discoloration reduction, the range of concentrations tested was limited. This leaves open the possibility that optimal concentrations exist that could offer even greater efficacy or that lower concentrations might suffice for significant effects, thereby reducing potential costs and increasing the accessibility of the treatment.

Furthermore, our study focused primarily on dentin’s aesthetic and physical properties following Zinc and SDF application. We did not explore the long-term effects of these treatments on oral health, including potential impacts on the microbiome, tissue response, or the durability of the treatment effect over time. These aspects are crucial for a holistic understanding of the implications of introducing Zinc into SDF treatment protocols.

### Future direction

The next steps will include conducting clinical trials to validate the effectiveness and safety of Zinc as an adjunct to SDF treatment in clinical settings. These studies should encompass a broader range of Zinc concentrations to identify the most effective and cost-efficient dosage for clinical use. Moreover, investigations into the long-term outcomes of Zinc-enhanced SDF treatments are essential. Future studies should examine the durability of the aesthetic improvements and potential impacts on caries development over extended periods, including monitoring changes in the oral microbiome and the recurrence of caries in treated teeth. Mechanistic studies are needed to understand how Zinc reduces discoloration and interacts with dental tissues, revealing the pathways involved. Additionally, patient-centered outcomes should be prioritized to evaluate how Zinc-enhanced SDF treatments influence the appearance and function of teeth from the patient’s perspective. Long-term safety and efficacy studies are critical to ensuring that Zinc continues to provide dental health benefits without adverse effects over time. Another compelling opportunity is to explore the potential synergistic effects of combining Zinc with other caries prevention strategies. For instance, examining how Zinc interacts with fluoride varnishes, dental sealants, or other antimicrobial agents could unveil new multidimensional approaches to caries management that leverage the strengths of each component.

## Conclusion

The study results reveal that Zinc significantly reduces SDF-induced tooth discoloration in a dose-dependent manner, offering a promising solution to one of the major aesthetic concerns associated with SDF treatment. Furthermore, the application of Zinc does not adversely affect the etched dentin’s hardness or roughness and oral mucosal cells, underscoring its potential as a safe and effective adjunct to SDF in caries management. Future research should focus on validating these findings in clinical settings and exploring the long-term safety and mechanistic interactions of Zinc-enhanced treatments.

## Data Availability

All data generated or analyzed during this study are included in this article. Further inquiries can be directed to the corresponding author.

## Clinical Significance:

Zinc-enhanced SDF treatments could enhance patient acceptance, especially in visible areas of the mouth, offering an improved option for caries management.
